# Laparoscopic repair of a Bochdalek hernia in an elderly patient: a case report with a review from 1999 to 2019 in Japan

**DOI:** 10.1186/s40792-020-01003-8

**Published:** 2020-09-29

**Authors:** Toshimitsu Miyasaka, Takeshi Matsutani, Tsutomu Nomura, Nobutoshi Hagiwara, Naoto Chihara, Koichi Takahashi, Keisuke Mishima, Nobuhiko Taniai, Hiroshi Yoshida

**Affiliations:** 1 Department of Gastrointestinal Hepato-Biliary-Pancreatic Surgery, Nippon Medical School, 1-1-5 Sendagi, Bunkyo-ku, Tokyo, 113-8603 Japan; 2 Department of Digestive Surgery, Nippon Medical School Musashikosugi Hospital, 1-396 Kosugimachi, Nakahara-ku, Kawasaki-shi, Kanagawa, 211-8533 Japan

**Keywords:** Bochdalek hernia, Adult diaphragmatic hernia, Surgery

## Abstract

**Background:**

A Bochdalek hernia (BH) is a congenital defect of the diaphragm that generally presents in the newborn as life-threatening cardiorespiratory distress. In contrast, the diagnosis of a BH in adults is rare. Surgical repair for adult BH is recommended, but the optimal surgical method remains unclear.

**Case presentation:**

A 75-year-old woman presented with progressive dyspnea and back pain, and a diagnosis of BH was made based on chest X-ray and computed tomography. Laparoscopic evaluation revealed a defect in the left posterior attachment of the diaphragm, and a left-sided BH without hernia sac was diagnosed. Parts of the stomach, small intestine, colon, pancreas, and spleen had prolapsed into the left thoracic cavity, without ischemic change, and these herniated organs were reduced to the abdominal cavity. A direct closure of the hernia orifice was possible by the laparoscopic suture technique using a mesh reinforcement. The patient made an uneventful recovery, and no recurrence was found in the 2-year follow-up.

**Conclusion:**

A recently published study reviewing detailed cases of repair of adult BH from 1999 to 2019 identified 96 cases, including the present case. The number of reports on laparoscopic and/or thoracoscopic surgery for BH in adults has recently increased, and the approach for repairing BH should be selected carefully on a case-by-case basis.

## Introduction

Most cases of Bochdalek hernia (BH) are diagnosed with severe respiratory and circulatory distress immediately after birth, and the mortality rate is high [1]. Presentation of BH during adulthood is relatively rare, with a reported frequency of 0.17–6% among all diaphragmatic hernias [1–6]. It is generally recommended that adults with BH undergo surgical repair to prevent life-threatening complications due to the incarceration of hernia, obstruction, strangulation, and perforation [7–9]. Surgical reduction of the prolapsed organs and the closure of the hernia orifice is required. Although recently published studies have described the efficacy of thoracoscopic and/or laparoscopic repair for BH in adults, there are currently no established optimal approaches or methods [2, 4, 10–15]. Herein, we describe our experience of adult BH in detail and review in light of surgical options from the available literature on Japanese cases.

## Case report

**Fig. 1:**
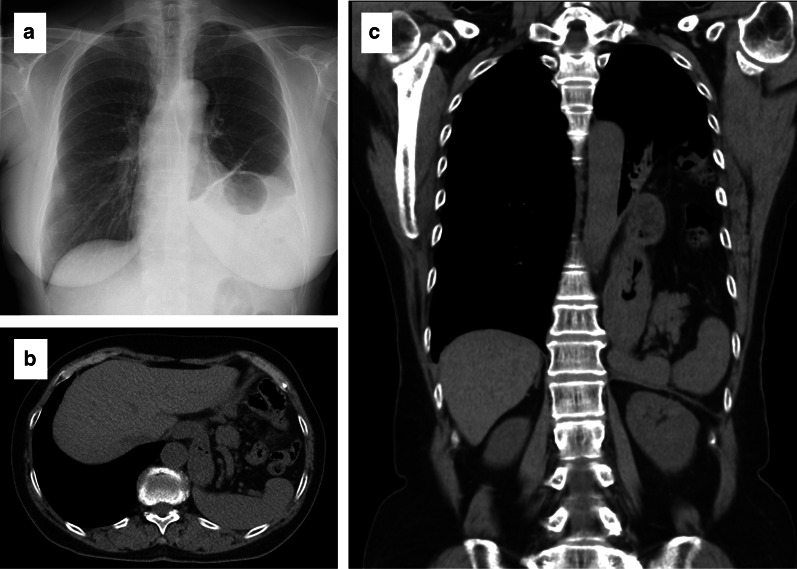
**a** Preoperative images of the Bochdalek hernia. The chest radiography on admission shows a lesion containing bowel gas in the left thoracic cavity. **b** Axial view images of the CT scan showing prolapse of the stomach, small intestine, colon, pancreas, and spleen into the left thoracic cavity. **c** Coronal view images of the CT scan showing same as above

**Fig. 2:**
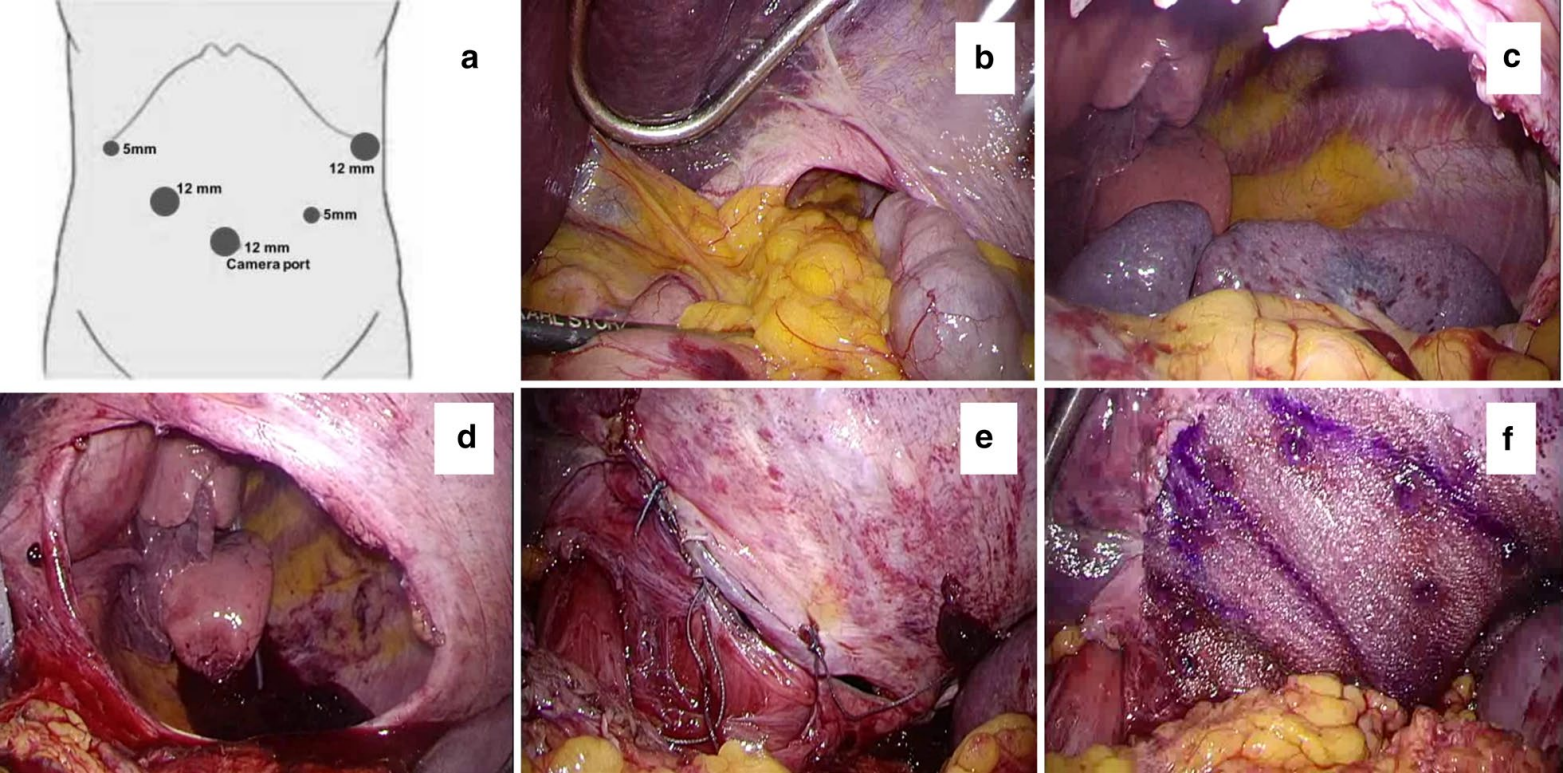
**a** Intraoperative views obtained upon laparotomy and port positions for abdominal access. **b** A large hernial orifice is observed in the left diaphragm, and the prolapsed stomach, small intestine, colon, and pancreas, are seen in the left thoracic cavity. **c** The spleen is entirely contained within the thoracic cavity due to adhesions. **d** Upon complete exposure, the hernial orifice is found to be 10 × 8 cm, without hernia sac. **e** The diaphragmatic defect is closed with interrupted nonabsorbable sutures. **f** The sutured site was reinforced with Ventralight™ ST mesh

A 75-year-old Japanese woman was admitted to hospital with the chief complaints of progressive dyspnea and back pain after eating, for 2 months prior. She had surgical histories of open appendectomy and laparotomy for bowel obstruction due to adhesions at 17 and 69 years old, respectively, but no histories of upper abdominal surgery or trauma. Upon physical examination, she measured 155.2 cm in height, weighed 53.0 kg, and had a BMI of 22.0 kg/m^2^. Her abdominal examination showed a lower abdominal median incision for bowel obstruction surgery and a right lower abdominal incision for appendectomy. The results of the laboratory tests were normal, but chest X-ray revealed an abnormal gas-filled bowel loops in the left thoracic cavity (Fig. 1a). Computed tomography (CT) revealed that the stomach, small intestine, colon, pancreas, and spleen had prolapsed into the left thoracic cavity, above the diaphragm, causing significant displacement of the left lung, which led to the diagnosis of a diaphragmatic hernia, such as para-esophageal hiatal hernia or BH (Fig. 1b, c). Liver was found on the normal right side. Laparoscopic hernia repair was performed under general anesthesia with endotracheal intubation by two-lung ventilation. Trocars were placed at the umbilicus (12-mm camera port), in the right upper abdomen (5-mm), at the right lower abdomen (12-mm port), left upper abdomen (12-mm port), and at the left lower abdomen (5-mm) (Fig. 2a). Pneumoperitoneum was established at 10 mmHg. When we examined the intra-abdominal cavity, we observed a large posterolateral defect of the left diaphragm. It was clear that part of the stomach, small intestine, transverse colon, spleen, and pancreas had prolapsed into the thoracic cavity, and there was no hernial sac (Fig. 2b). These hernia contents were reduced back into the abdominal cavity from the left thoracic cavity with the use of atraumatic forceps, and no ischemic change was identified in any of the organs. However, the spleen was entirely contained within the thoracic cavity. The reduction of the spleen into the abdominal cavity was difficult, because the gastrosplenic ligament had adhered to the parietal pleura (Fig. 2c). The adhesions were exfoliated using an ultrasonic coagulator. Thereafter, the spleen was carefully placed back into the abdominal cavity. Upon examination of the thoracic cavity, no pulmonary adhesions were observed, and a left posterolateral diaphragmatic defect measured approximately 10 × 8 cm in diameter (Fig. 2d). The diaphragmatic defect was closed using a single layer primary closure method with nonabsorbable 2-0 Ethibond interrupted sutures (Fig. 2e). Because the diaphragm around the hernial orifice was somewhat fragile, a Ventralight™ ST mesh, coated with a chemically modified substance (Bard/Davol, Warwick, USA), was placed over the defect (Fig. 2f). The mesh was fixed to the diaphragm with an absorbable tacker. We did not leave a drain in the left chest cavity. The collapsed lung could be re-expanded by positive pressure ventilation without developing acute lung edema. The surgery lasted 266 min, and the blood loss volume was 215 ml. The postoperative course was uneventful and the patient was discharged in good condition on postoperative day 7. There was no recurrence in the 27 months after the surgery.

## Discussion

As in Western and other countries, in Japan, adult BH is an uncommon form of diaphragmatic hernia. To date, the optimal evaluation technique and treatment strategy for adult BH remain to be established. We reviewed the Japanese reported experience in order to assess the effectiveness of the surgical treatments. A review of the literature published from January 1999 through December 2019 was performed by searching the PubMed database and the Ichushi-Web database of the Japanese Medical Abstract Society (https://login.jamas.or.jp/; NPO Japan Medical Abstracts Society) using the following key word: “Bochdalek hernia.” Case reports and reviews of case reports with full texts or abstracts were included. Further cases were identified by cross-referencing the references that were cited in other case reports and reviews. All patients aged 20 years and above were considered as adults and were included in this study. To examine trends in BH surgery, only reports from Japan, where a national health insurance system is established, were included in this study. The electronic literature search yielded 5,510 (PubMed database: 5,229, Ichushi-Web database: 281) hits. Of these, we included 107 studies based on title and abstract review; 13 were excluded from the manual search. The 94 remaining case studies, published between 1999 and 2019, matched our inclusion criteria for this analysis. We accessed and analyzed 96 case reports of adult BH including the present case (Table 1) [10–15].

**Table 1:** Characteristics of 96 Japanese cases of Bochdalek hernias in adults, literature review (1999 − 2019)

	No. of patients 96
Age at diagnosis of adult Bochdalek hernia (years)	
Median (years)	58
Range (years)	20 − 89
Sex (male/female)	38/58
Hernia orifice location	
Right/left/NA	18/73/5
Size of the hernia orifice	
< 5 cm/≧ 5 cm/NA	21/40/35
Presenting symptoms	
Asymptomatic	6
Abdominal symptoms	65
Abdominal pain	56
Back pain	5
Abdominal distension	4
Pulmonary symptoms	28
Dyspnea	19
Chest pain	11
Obstructive symptoms	42
Vomiting	31
Nausea	11
Dysphagia	3
Others	17
Hernia contents	
Stomach	46
Colon	40
Omentum	27
Small intestine	24
Spleen	20
Liver	6
Pancreas	4
Kidney	4
NA	5

*NA* not available

In terms of background, adult BH has a female predominance, and 58 patients (60%) were female, with a mean age of 58 years (range 20–89 years); 54 patients (56%) were above 60 years and 28 (29%) were aged 70–80 years. These findings may indicate that adult BH occurs more frequently in elderly persons. The left/right side ratio of adult BH in these cases was 73:18, but the details were unreported in 5 patients; this ratio is similar to that in previous studies [1, 2]. The hernia contents were the stomach (*n* = 46), colon (*n* = 40), omentum (*n* = 27), small intestine (*n* = 24), spleen (*n* = 20), liver (*n* = 6), pancreas (*n* = 4), kidney (*n* = 4), and retroperitoneal tissue (*n* = 5). The most common initial presentations of adult BH were abdominal (pain, distension, discomfort; 67.7%), pulmonary (cough, chest pain, dyspnea; 29.1%), and obstructive symptoms (vomiting, nausea; 43.8%). Therefore, adult BH should form one of the differential diagnoses in patients who present with simultaneous abdominal and chest symptoms. In contrast, 6 (6.7%) cases were asymptomatic. Furthermore, the most severe complications of adult BH were incarcerated, gastric volvulus, and intestinal perforation, which were reported in 17 (17.7%) of cases. Accurate diagnosis of adult BH is difficult because of its atypical symptoms and variation in hernia content [4, 5]. Indeed, the misdiagnosis rate of adult BH has been reported to be as high as 38% [2, 16], and misdiagnosis of adult BH may lead to inappropriate sudden death [7].

**Fig. 3:**
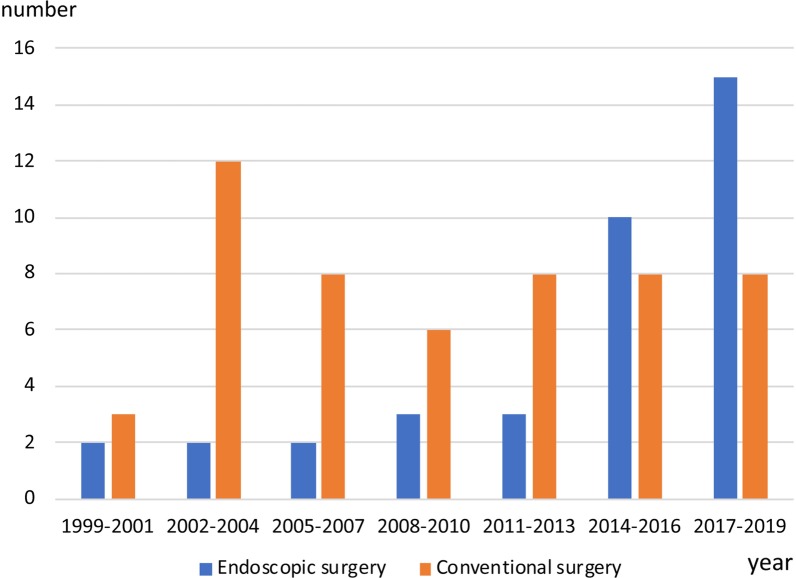
Trends in the numbers of surgeries for adult Bochdalek hernia from 1999 to 2019 grouped by operative method (endoscopic versus conventional surgery)

Surgical reduction of the prolapsed organs and closure of the hernial orifice are recommended immediately following diagnosis/suspicion of adult BH [4, 7]. Transthoracic (thoracotomy), transabdominal (laparotomy), and combined thoracoabdominal routes are the open (conventional) approaches. Recently, the number of reports of thoracoscopic and/or laparoscopic surgery for treatment of adult BH has increased (Fig. 3). However, there is no established consensus for the selection of an approach or surgical method. A published study that reviewed the detailed cases of laparoscopic and/or thoracoscopic repair of adult BH from 1999 to 2019 identified 37 Japanese cases (Table 2). The mean age of the patients was 54 (20–87) years, and the male/female sex ratio was 17/20. The approaches were laparoscopic (*n* = 23), hand-assisted laparoscopic (*n* = 4), thoracoscopic (*n* = 6), and combined thoraco-laparoscopic repair (*n* = 4). In contrast, conventional surgical approaches were selected for 53 patients, 18 men and 35 women, with a mean age 59 (21–89) years, including laparotomy, thoracotomy, and combined thoracoabdominal approach in 45, 4, and 4 patients, respectively. A conversion to laparotomy from laparoscopic approach was required in 5 cases. However, the remaining 6 (8.7%) patients did not undergo any surgical procedure (chest drainage, *n* = 2; conservative therapy, *n* = 4). Among the emergency cases with bowel perforation, all 12 (100%) patients were treated by the conventional approach as follows: laparotomy (*n* = 9), thoracotomy (*n* = 1), and combined thoracoabdominal approach (*n* = 2).

**Table 2:** Characteristics of Japanese cases with endoscopic and conventional surgery for adult Bochdalek hernia, literature review (1999 − 2019)

	Endoscopic surgery	Conventional surgery
All cases	37	53
Age at diagnosis (years)		
Median (years)	54	59
Range (years)	20−87	21−89
Sex (male/female)	17/20	18/35
Operative approach		
Transabdominal	27	45
Transthoracic	6	4
Combined	4	4
Hernia orifice location		
Right/left/NA	2/32/3	14/37/2
Size of the hernia orifice		
< 5 cm/≧ 5 cm/NA	7/19/37	13/20/20
Hernia sac		
Present/absent/NA	5/14/18	3/27/23
Abnormal condition		
Bowel rotation	4	5
Bowel perforation	0	12
Hernia orifice repair		
Simple suture	13	38
Suture and mesh	13	3
Mesh only	7	5
No repair	4	3
NA	7	4

*NA* not available

Direct suture closure of the hernial orifice during hernia repair was generally performed in 41 patients (77%) who underwent conventional repair: simple sutures (*n* = 38) and sutures and mesh (*n* = 3). Among the remaining 12 patients, mesh only (*n* = 5) or no repair (*n* = 3) was selected, but the details were unreported in 4 patients. In those with laparoscopic repair, the hernia orifice was repaired with simple sutures (*n* = 13), sutures and mesh (*n* = 13), mesh only (*n* = 7), no repair (*n* = 4), and by an unknown means (*n* = 7). Mesh reinforcement was required in more than 50% of patients undergoing laparoscopic repairs, compared to only 15% of patients who underwent conventional repairs. In the present case, suturing and mesh reinforcement were performed when the tissues around the hernia orifice were fragile. We selected the direct closure with nonabsorbable sutures and Ventralight™ ST mesh, which is coated with chemically modified sodium hyaluronate and carboxymethylcellulose, reinforcement to minimize adhesions [17]. This review notes that most of the patients had satisfactory clinical courses without hernia recurrence, but one patient died as a result of colon perforation, pneumonia, and sepsis.

## Conclusion

The number of endoscopic surgeries for adult BH has increased as a result of improvements in techniques and devices. However, the approach to BH repair needs to be selected carefully on a case-by-case basis.

## Data Availability

The data are not available for public access due to patient privacy concerns but are available from the corresponding author on reasonable request.

## Abbreviations

BH: Bochdalek hernia
CT: Computed tomography
